# A software pipeline for systematizing machine learning of speech data

**DOI:** 10.3389/fpsyt.2025.1451368

**Published:** 2025-07-29

**Authors:** Jimuel Celeste, Mashrura Tasnim, Amable J. Valdés Cuervo, Enrique A. de la Cal, Eleni Stroulia

**Affiliations:** ^1^ Department of Computing Science, Faculty of Science, University of Alberta, Edmonton, AB, Canada; ^2^ Computer Science Department, Faculty of Geology, University of Oviedo, Oviedo, Spain

**Keywords:** speech analysis, digital mental health, depression, dementia, aphasia, machine learning for speech audio, software pipeline for speech signal processing

## Abstract

The reproducibility and replicability of experimental findings is an essential element of the scientific process. The machine-learning community has a long-established practice of sharing data sets so that researchers can report the performance of their models on the same data. In the area of speech analysis, and more specifically speech of individuals with mental health and neurocognitive conditions, a number of such data sets exist and are the subject of organized “challenge tasks”. However, as the complexity of the available relevant software libraries and their parameters increases, we argue that researchers should not only share their data but also their preprocessing and machine learning configurations so that their experiments may be fully reproduced. This is why we have designed and developed a suite of configurable software pipelines with Python Luigi for speech-data preprocessing, feature extraction, fold construction for cross-validation, machine learning training, and label prediction. These components rely on state-of-the-art software libraries, frequently used by researchers, and implement many typical tasks in this field, i.e., scikit-learn, openSMILE, LogMMSE, so that, given the configuration parameters of each task, any underlying experiments can be readily reproduced. We have evaluated our platform by replicating three different machine learning studies, with the aim of detecting depression, mild cognitive impairment, and aphasia from speech data.

## 1 Introduction

The global burden of mental health disorders and cognitive decline is substantial, with statistics indicating a pressing need for innovative solutions. According to the World Health Organization (WHO), depression affects over 280 million people worldwide (1), while Alzheimer’s disease and other forms of dementia impact an estimated 55 million individuals (2). Moreover, the prevalence of these conditions is expected to rise significantly in the coming years, posing challenges to healthcare systems worldwide (3). Consequently, there is a growing demand for digital solutions that could enable more timely and accessible support to those in need. In response to these challenges, researchers have increasingly turned to speech and vocal tone-oriented systems, empowered by advances in artificial intelligence and machine learning, as promising avenues for digital monitoring and early assessment of mental health conditions and cognitive impairment when treatment is more effective (4–8).

Experiment reproducibility and replicability are challenges in machine learning (ML) research. Reproducibility is defined as the ability to obtain the same precise results of an experiment with identical conditions (code and configuration) and data. Replicability refers to getting similar result trends and conclusions, given that conditions remain identical even if the data is different. Both concepts are important in ensuring that findings are verifiable and generalizable. However, analysis shows that ML research suffers from what is termed the ‘reproducibility crisis’ (9, 10).

Key challenges identified include the lack of detailed documentation, restricted access to code and data, and the innate randomness of machine-learning methods (9–12). This crisis is acknowledged by the research community at large. An analysis of INTERSPEECH Conference publications in 2023 (11) found that only 40% of the papers in this venue were published with artifacts (code and data) and recommended that authors should publish such artifacts and event organizers should establish reproducibility requirements, similar to Neural Information Processing Systems (NeurIPS) which introduced a reproducibility program in 2019. They observed that the number of works published with artifacts increased and such artifacts were consulted in reviewing submissions (12).

In addition, data leakage, inadequate model validation, and exaggerated claims are among the reasons cited for why some machine learning studies are more difficult to reproduce (13). As of May 2024, the “Leakage and the Reproducibility Crisis in ML-based Science” project^5^ at Princeton’s Center for Information Technology Policy^10^ (14) has curated a list of 41 papers from 30 fields where errors have been found, collectively affecting 648 papers and in some cases leading to wildly overoptimistic conclusions.

Our work aims to improve the reproducibility and replicability in speech-analysis experiments through sharing a different type of artifact. Code repositories are often difficult to reuse due to their dependencies on third-party libraries. As the field evolves, feature sets are being standardized, and machine-learning algorithms are established as benchmarks. Therefore, we have developed a modular and configurable set of pipelines to perform audio preprocessing, feature extraction, machine learning training, and predicting the prevalence and severity of certain mental and cognitive disorders, where each module encapsulates well-known algorithms (6, 15–17), explicitly configurable through the algorithm parameters. Whereas replicating experiments through shared code is tedious, requiring code and environment adaptations to resolve hard-coded data paths and dependencies, replicating an experiment through our pipeline is simply a matter of selecting the appropriate algorithms and configuring them with the appropriate parameters. In this manner, experiments become better documented and easily studied by writing configuration files and running pipelines with any valid dataset.

There are two similar works aiming at advancing reproducibility and replicability in speech analysis. VoiceLab (18) is a software designed for audio manipulation, analysis, and visualization. It supports amplitude normalization in addition to a wide array of preprocessing functionalities currently not included in our work. However, audio denoising is not currently supported by VoiceLab, as well as machine learning tasks. TRESTLE (19), on the other hand, had been designed to extract linguistic and acoustic features from transcripts and audio recordings, respectively. Its functionalities include audio format conversion, audio resampling, Fourier Transformation, and MFCC feature extraction. Again, TRESTLE does not currently support machine-learning tasks.

Compared to previous work, our pipeline advances the state of the art in machine learning for speech in two important dimensions. First, our pipeline supports a broader variety of features, based on openSMILE (20) and openXBOW (21). Second, whereas these tools focus on supporting data preprocessing and feature extraction, our work covers the complete machine-learning workflow, including data preprocessing, experiment design and cross-validation, training machine-learning models, and using them for prediction.

## 2 Materials

This section describes three datasets used in conducting experiments to evaluate our software pipeline for training ML models aimed at predicting three prevalent mental and neurocognitive disorders: depression, mild cognitive impairment, and aphasia. **Table 1** summarizes some key aspects of the datasets used in the experiments.

**Table 1:** Description of the three datasets used in the experiments.

Property	AVEC 2013	TAUKADIAL 2024	PPA pilot study
Mental Health Condition	Depression	Mild Cognitive Impairment (MCI)	Primary Progressive Aphasia (PPA)
Machine Learning Task	Regression	Regression and Classification	Classification
Language	German	English and Chinese	Spanish
Speech Task	Guided reading and freeform speech	Picture description	Verbal fluency test, work and sentence repetition, and picture naming
Cognitive Assessment Tool (Range)	Beck Depression Index-II (0-63)	Mini-Mental State Examination (0-30)	Expert diagnosis
# Subjects	83	129	12
Average Length of Samples	< 5 minutes	1 minute	6.69 minutes
# Cross-Validation Samples	300	387	246
Sex Distribution	no sex information	237 women; 150 men	0 woman; 246 men
Subject-Wise Mean Age (Standard Deviation)	31.5 (±12.3)	72.7 (±6.4)	71.8 (±4.8)
Sample-Wise Mean Age (Standard Deviation, Range)	31.5 (12.3, 18-63)	MCI: 73.36 (6.14, 61-87) NC: 71.85 (6.65, 61-87)	71.83 (4.80, 65-79)
Distribution of Samples	None (0-13): 154 Mild (14-19): 44 Moderate (20-28): 52 Severe (29-63): 50	MCI: 222 Normal Control (NC): 165	PPA: 127 NC: 119
Average Score (Standard Deviation)	15.1 (12.3)	MCI: 25.84 (3.73) NC: 29.07 (1.07)	–

The AVEC 2013 depression dataset (4) consists of recordings from 84 subjects, between 18 and 63 years old (mean=31.5(± 12.3)). Every participant was recorded while 1) reading aloud a part of the fable "The North Wind and the Sun," and 2) while answering one question, including "what is your favorite dish," "what was your best gift, and why", and "discuss a sad childhood memory"; both tasks were in German. The recordings were divided into three partitions: a training, development, and test set of 150 Northwind-Freeform pairs. In our work, we used only the training partition. The recordings were labeled with the Beck Depression Index (BDI) of the speakers. The scores range from 0 to 63, where 0 indicates minimal depression and 63 indicates severe depression. The highest BDI score in this dataset is 45, which may affect the performance of the proposed system in real-world scenarios.

The INTERSPEECH 2024 TAUKADIAL challenge dataset consists of Chinese and English speech samples collected while the speakers describe a picture, as part of a cognitive assessment protocol (8). English-speaking participants completed the discourse protocol and cognitive-linguistic battery, guided by a facilitator. The discourse protocol tasks include three picture-description tasks: 1) the "Cookie Theft" picture (22); 2) the "Cat Rescue" picture (23); and 3) the Normal Rockwell print "Coming and Going" (24). Chinese-speaking participants described a set of three pictures depicting scenes from Taiwanese culture. For both languages, the participants with MCI were diagnosed by experts in neuropsychology, according to the National Institute on Aging-Alzheimer’s Association (NIA-AA) (25). In this work, we used the 387 training samples to perform binary classification of MCI (mild cognitive impairment) or NC (normal control).

In (26), a combination of cognitive tests (Addenbrooke’s Cognitive Examination III (ACE-III) (27), Mini Linguistic State Examination (MLSE) (28), and BETA (29)) were used to assess potential PPA patients. This PPA-Tool collects a larger corpus of speech recordings across most oral production cognitive areas than individual cognitive tests like ACE-III and MLSE. The earlier work analyzed the recordings from the first group of participants, which included 6 Spanish speakers (4 with PPA and 6 controls). The dataset used in our current experiments include 12 Spanish speakers (6 with PPA and 6 controls). This PPA pilot study focused on the following tasks for the machine learning analysis: 1) Fluency: the verbal fluency tasks from ACE-III; 2) Repetition: the repetition of words and sentences from the three tests; and 3) Naming: the picture naming task from MLSE. The verbal fluency task consists of three subtests: phonological, semantic, and actions. In these tasks, participants generate as many words as possible within one minute for each category (words beginning with “p”, animals, and actions, respectively). The picture naming test involves naming 20 images presented to the participant. Finally, the repetition task includes both word and sentence repetition. After removing silence, the complete study lasted 27 mins, distributed as follows: Fluency 37%, Repetition 39%, and Naming 24%.

## 3 Method

In this section, we describe our software pipeline and detail the experiments carried out to validate it. We demonstrate its effectiveness by applying it to three datasets described in Section 2.

### 3.1 The software architecture

**Figure 1:**
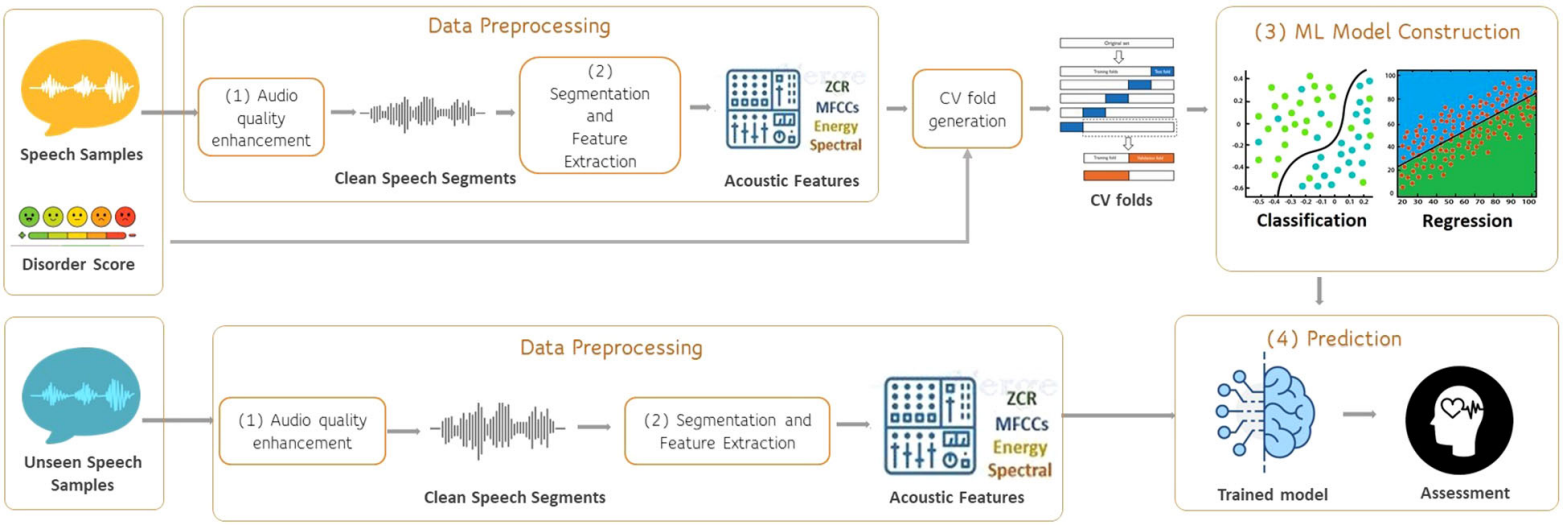
Software pipeline for speech processing to infer mental health.

Our team took part in the challenges, conducting experiments on the datasets mentioned above. In this context, we have developed an extensive code base and substantial experience of the important algorithmic decisions involved in analyzing speech for determining mental and cognitive health indicators. To consolidate this code and experience so that they can benefit new researchers, we have developed a software system that makes the possible computational tasks explicit in speech processing for mental health inference. **Figure 1** depicts the overall methodology for analyzing speech data to construct and evaluate machine learning models, showcasing the two main pipelines we have implemented, 1) data preprocessing and feature extraction and 2) machine learning training. Prediction is simply a composition of the data-preprocessing and feature extraction pipeline and the invocation of the learned machine-learning model.

#### 3.1.1 Luigi

Our software was implemented with Luigi^1^ a workflow management framework in Python, developed by Spotify. Luigi models workflows as directed acyclic graphs where nodes represent tasks and edges represent dependencies. Tasks are processes that have input/s, output/s, and an implementation process. The dependencies between tasks are modeled by feeding the output of one task as input for another. This modeling allows the construction of complex pipelines. When running the pipelines, Luigi automatically manages various background processes, including task scheduling, dependency resolution, redundancy avoidance, and failure recovery. In addition, Luigi provides a dashboard which tracks the status of tasks.

#### 3.1.2 Data preprocessing and feature extraction

The tasks included in the preprocessing and feature extraction pipeline and their corresponding parameters are detailed in **Table 2**. Running the pipeline requires three inputs: 1) the directory containing the input data, 2) the directory where the output will be stored, and 3) the configuration file. The pipeline runs each and every task specified in the configuration file for all the input files inside the input directory. It then saves all preprocessing artifacts in the output directory.

**Table 2:** Parameters of the preprocessing and feature extraction pipeline.

Task	Task name	Parameters
Audio Format Conversion	convert format	input format: input file format, e.g., “mp4”, “mp3” output format: target output file, e.g., “wav”
Audio Bit-depth Conversion	convert bit depth	bit depth: target bit depth
Denoising	denoise	initial noise: (by default, 6)window size: (by default, 0)noise threshold: (by default, 0.15)
Amplitude Normalization	normalize amplitude	target dbfs: target decibel relative to full scale (dBFS, by default=-20).
openSMILE Feature Extraction	opensmile	feature set: the feature set to be extracted level: the feature level to be extracted
openXBOW Feature Extraction	openxbow	audio book size: number of patterns to search for openxbow jar path: path to the openXBOW jar instance

Data preprocessing is composed of four steps: 1) audio format conversion, 2) audio bit-depth conversion, 3) denoising, and 4) amplitude normalization. Audio format conversion converts audio files from one format to another. This task was included in the pipeline since the succeeding tasks require wav format for the audio files. This task is performed with pydub’s AudioSegment module^2^. It supports all file formats supported by ffmpeg^3^. Audio bit-depth conversion converts audio files from one bit-depth type to another. This task was included as a prerequisite for the denoising task, which only supports audio files with bit-depth “32-bit floating-point, 32-bit PCM, 16-bit PCM, and 8-bit PCM”^4^. Bit-depth conversion is performed with soundfile^5^. Denoising is done with logmmse^6^. For amplitude normalization, the apply_gain function of pydub’s AudioSegment module is used. It normalizes the amplitude of the input audio file with a specified target dBFS.

After the above preprocessing data transformations, feature extraction is performed with openSMILE (20) and openXBOW (21). The wide variety of features that can be extracted with openSMILE is detailed in **Table 3**. openXBOW computes Bag-of-Audio-Word (BoAW) features on low-level descriptors (LLDs) extracted with openSMILE.

**Table 3:** Features that can be extracted with openSMILE.

Feature Set	Level		
	Low-Level Descriptors	Functionals	LLD Deltas
ComParE 2016	Yes	Yes	Yes
GeMAPSv01a	Yes	Yes	No
GeMAPSv01b	Yes	Yes	No
eGeMAPSv01a	Yes	Yes	No
eGeMAPSv01b	Yes	Yes	No
eGeMAPSv02	Yes	Yes	No

#### 3.1.3 Training machine learning models

Typically, k-fold cross-validation is performed to select the optimal set of hyperparameters for machine learning models. The parameters for this task are described in **Table 4**. First, folds are generated with scikit-learn’s StratifiedGroupKFold module^7^. This module ensures that all samples from the same individual belong only to one partition of a fold, i.e., subject-wise cross-validation. All segments generated from one sample also belong to one partition in each fold.

**Table 4:** Parameters of the machine learning model training pipeline K-fold cross validation task.

Sub-tasks	Parameters
Fold Generation	folds: number of folds for K-fold cross-validationrandom state: random seed for fold generation shuffle: shuffle samples or not (boolean)
Model	model task: ‘classification’ or ‘regression’ estimator: ‘svm’ or ‘random forest’estimator parameters: parameters of themodel estimator
Parameter Grid	hyperparameters to optimize
Feature Selection	k percentage: percentage of the number of features to retain
Performance Evaluation	metrics: ‘accuracy’, ‘precision’, ‘recall’, and ‘f1’ for classification; ‘mae’, ‘mse’, ‘rmse’, and ‘r2’ for regression.

To date, our software supports two machine-learning algorithms for the classification and regression tasks in the machine-learning pipeline: Support Vector Machine, a common baseline model (21, 30, 31), and Random Forest, an algorithm that has been shown to perform competitively in the literature. Expanding the algorithm set would require additional implementations for these tasks.

The machine-learning pipeline invokes six sub-tasks:

1. Features are standardized with scikit-learn’s StandardScaler^^8^^;
2. Feature selection is performed with scikit-learn’s SelectKBest^^9^^;
3. Hyperparameters are tuned with an internal 5-fold grid search using scikit-learn’s GridSearchCV^^10^^;
4. Model is retrained with the optimized hyperparameter and the training folds;
5. The optimized model is validated with metrics specified by the user; and
6. Optimized models are saved as Python pickle files ^^11^^.

This pipeline has three inputs: 1) data, 2) output directory, and 3) configuration. The input data is expected as a csv file containing the columns id, group, label, and features. Column id pertains to the unique sample id, while group pertains to the unique subject id. Similar to the preprocessing and feature extraction pipeline, this pipeline runs all the tasks specified in the configuration with the data provided, then saves all artifacts in the output directory.

The artifacts of the k-Fold cross-validation task, for each fold, are as follows: 1) validation predictions, 2) selected features, 3) optimal hyperparameters, 4) model, and 5) model performance. These artifacts could be analyzed to investigate the performance of the models, for instance, in error analysis.

#### 3.1.4 The pipeline configuration manifest

The two pipelines can be configured with user-defined YAML configuration manifests; as shown in **Tables 5**–**7**. A configuration manifest is composed of a name, a description, and a set of tasks. Each task is defined by a name, a unique id, an input id, and its parameters. The input id is the unique id of another task, allowing users to link one task to another. For tasks that require raw inputs, keyword ‘input’ is reserved as the input id.

**Table 5:** Sample preprocessing and feature extraction configuration file.

name: Sample Preprocessing and Feature Extraction Config description: 1) Bit-depth conversion, 2) Denoising, 3) Amplitude Normalization, 4) openSMILE feature extraction, 5) openXBoW feature extraction pipeline: - task: convert_format id: wav_files input_id: input parameters: input_format: mp4 output_format: wav - task: convert_bit_depth id: 16bit_wav input_id: wav_files parameters: bit_depth: PCM_16 - task: denoise id: denoised_audio input_id: 16bit_wav parameters: initial_noise: 6 window_size: 0 noise_threshold: 0.15 - task: normalize_amplitude id: normalized_audio input_id: denoised_audio parameters: target_dbfs: -20 - task: opensmile id: compare_2016_lld input_id: normalized_audio parameters: feature_set: compare_2016 level: lld - task: openxbow id: boaw_size_500_compare_2016_lld input_id: compare_2016_lld parameters: openxbow_jar_path: 'path/to/openxbow.jar' audio_book_size: 500

**Table 6:** Sample classification model training configuration file.

name: Sample Classification Model Training Config description: Leave-One-Group-Out Cross-Validation (12 speakers) pipeline: - task: cross_validation id: svm_classification input_id: input parameters: fold_generation: folds: 12 random_state: 50 shuffle: True model_training: model: task: classification estimator: svm estimator_parameters: kernel: rbf parameter_grid: C: [0.1, 1, 10, 100] gamma: [1, 0.1, 0.01, 0.001, 'scale'] max_iter: [50, 100, 150] feature_selection: k_percentage: 1 model_evaluation: metrics: ['accuracy', 'precision', 'recall', 'f1']

**Table 7:** Sample regression model training configuration file.

name: Sample Regression Model Training Config description: 20-fold Cross-Validation pipeline: - task: cross_validation id: random_forest_regression input_id: input parameters: fold_generation: folds: 20 random_state: 50 shuffle: True model_training: model: task: regression estimator: random_forest estimator_parameters: random_state: 42 parameter_grid: n_estimators: [50, 100, 150, 200] max_features: ['log2', 'sqrt'] max_depth: [5, 10, 15] feature_selection: k_percentage: 1 model_evaluation: metrics: ['mae', 'mse', 'rmse', 'r2']

## 4 Experimental evaluation and results

We evaluated our software platform by replicating three studies, reported in (4, 6, 8, 26). It is important to note that the second study (6) was led by the second author of this paper, who was the lead developer of most of the code artifacts used in the software. The third study was conducted independently by the third and fourth authors and their code artifacts.

### 4.1 Experimental setup

The experiments aim to provide baseline model performance with standard feature sets. The experiments included four feature sets and two models. The four feature sets are ComParE 2016 functionals, GeMAPS functionals, eGeMAPS functionals, and Bag-of-Audio-Words extracted from ComParE 2016 LLDs. These are standard feature sets reported in the literature (8, 20, 21). The two models are Support Vector Machine (SVM) and Random Forest (RF). We chose the algorithms used in the two studies we replicated, i.e., AVEC 2013 baseline (4, 6) and the PPA study (26). In this manner, we demonstrate the ability of our software pipeline to replicate experimental work. AVEC 2013 baseline results were reported for SVM and Random Forest, and the first PPA study reported good performance on tree-based models, i.e., Random Forest.

We ran the experiments on a MacBook Air with Apple M1 chip (8 CPU cores) at a clock speed of 3.2 GHz, 8 GB memory, and Sequoia version 15.3.1 operating system. The 8 CPU cores of the system were utilized during model training by setting GridSearch’s n_job parameter to -1. The Luigi pipelines support multiprocessing by setting a number of workers to run multiple tasks in parallel. We however did not utilize this functionality in the experiments we performed

#### 4.1.1 Data preprocessing and feature extraction

There are 201 WAV files in the TAUKADIAL dataset with bit-depth PCM-16, while the rest (186 files) were in PCM-24. Therefore, the preprocessing included bit-depth conversion to PCM 16 before denoising and amplitude normalization. The denoising and amplitude normalization tasks were configured with the parameters detailed in **Table 5**. The four feature sets were then extracted from the preprocessed audio files. For BoAW features, audio_book_size=500 features were extracted.

The AVEC 2013 dataset underwent the same preprocessing, with an additional MP4-to-WAV conversion step. The same four feature sets were extracted, including BoAW features with audio_book_size=500.

The PPA pilot study dataset followed the TAUKADIAL preprocessing pipeline. Again, four feature sets were extracted, but for BoAW features, audio_book_size=100 was used due to the dataset’s shorter samples.

#### 4.1.2 Machine learning

We constructed machine-learning models for a number of combinations of learning tasks and feature sets. The TAUKADIAL challenge presents two tasks: 1) binary classification between individuals with mild-cognitive impairment (MCI) and normal controls (NC); and 2) regression for their corresponding MMSE scores. The AVEC 2013 challenge presents a regression task for BDI scores. The PPA tool presents a binary classification task for PPA vs healthy individuals. A total of 16 experiments were conducted:

1. 2 tasks × 4 feature sets = 8 training sets for TAUKADIAL dataset
2. 1 task × 4 feature sets = 4 training sets for AVEC 2013 dataset
3. 1 task × 4 feature sets = 4 training sets for PPA pilot study dataset

For each of these experiments, SVM and RF models were trained. The model parameters were tuned using internal K-Fold cross-validation, detailed in **Tables 6** and **7**. For the TAUKADIAL 2024 and AVEC 2013 datasets, a 20-fold CV was implemented. For the PPA pilot study dataset, a 12-fold CV was conducted, equivalent to a leave-one-patient (group)-out framework, since there are 12 subjects (6 PPA, 6 healthy) in total. No feature selection was performed for the experiments.

We assessed the performance of the trained classifiers using the unweighted average recall (UAR) (Equation 1) and F1 score (F1) (Equation 2) metrics:


(1) $$UAR=\frac{\sigma +\rho }{2}$$

and


(2) $$F1=\frac{2\pi \rho }{\pi +\rho }$$

Here, *σ* is specificity (Equation 3), *ρ* is sensitivity (Equation 4), and *π* is precision (Equation 5):


(3) $$\sigma =\frac{T_{N}}{T_{N}+F_{P}}$$

(4) $$\rho =\frac{T_{P}}{T_{P}+F_{N}}$$

(5) $$\pi =\frac{T_{P}}{T_{P}+F_{P}}$$

where N is the total number of samples, $T_{P}$ is the number of true positives, $T_{N}$ is the number of true negatives, $F_{P}$ is the number of false positives, and $F_{N}$ is the number of false negatives.

The regression models were evaluated using root mean squared error (RMSE) (Equation 6), calculated as:


(6) $$\text{RMSE}\left(y,\hat{y}\right)=\sqrt{\frac{\sum_{i=1}^{N}{\left({\hat{y}}_{i}-y_{i}\right)}^{2}}{N}}$$

where y and $\hat{y}$ represent the ground truth and the predicted scores on ith sample, and N indicates the total number of samples.

As part of the machine-learning model construction, the pipeline implements a variety of statistical measures, to evaluate the performances of the trained models. For this study, we calculated the mean, standard deviation, and 95% confidence interval (CI) of the cross-validation results. To determine if there are statistically significant differences in the performances of the best performing classification and regression models, we performed McNemar Test and ANOVA, respectively. For models with statistically significant difference, we reported Cohen’s d.

### 4.2 Findings

**Figure 2:**
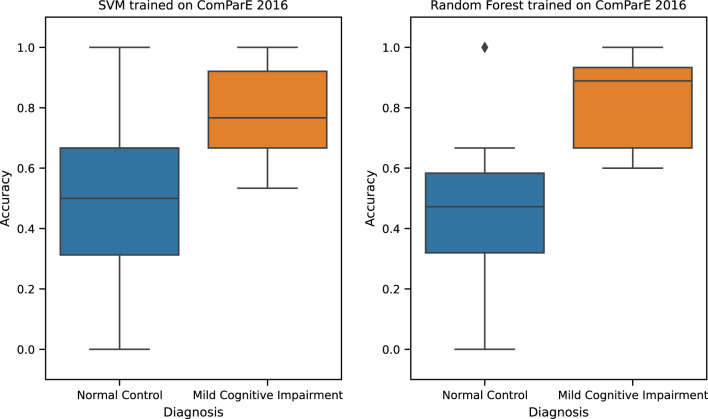
TAUKADIAL 2024 best classification models accuracy.

The best performing TAUKADIAL classification model (**Table 8**) is Random Forest trained with ComParE 2016 functional features. This model has a reported UAR of 0.66 (±0.13) [0.60, 0.73] (in brackets is the 95% CI). This performance is same as the baseline performance reported in (8) with a UAR score of 0.66 (no standard deviation reported) [0.63, 0.73]. The only difference is the slightly narrower 95% CI of the latter. Similarly, close to Random Forest’s performance is SVM trained with ComParE 2016 functional features. This model yields a UAR score of 0.65 (±0.17) [0.57, 0.73]. The difference between the two models is small with effect size of *d* = 0.10 (*p* < 0.05). Hence, we consider the two models to not be very different in terms of performance. Error analysis of these two best-performing models reveals that they are biased against the Normal Control (NC) class (see **Figure 2**). The model is more likely to misclassify samples from NC class than the Mild Cognitive Impairment (MCI) class, thus producing more false positives. The computed median accuracy is higher and the distribution is less dispersed on the MCI than the NC class, suggesting performance stability for the former. This analysis implies the need for a way to address the performance deficit of the model on the NC class to improve its overall UAR.

**Table 8:** TAUKADIAL 2024 classification cross-validation results (*k* = 20).

Feature Set	SVM		Random Forest	
	UAR (± std)	95% CI	UAR (± std)	95% CI
ComParE 2016 Functionals	0.65 (0.17)	[0.57, 0.73]	0.66 (0.13)	[0.60, 0.73]
GeMAPS Functionals	0.55 (0.12)	[0.49, 0.60]	0.60 (0.16)	[0.53, 0.68]
eGeMAPS Functionals	0.59 (0.15)	[0.52, 0.66]	0.61 (0.14)	[0.55, 0.68]
Bag of Audio Words (n=500)	0.47 (0.14)	[0.41, 0.54]	0.51 (0.10)	[0.47, 0.56]
Challenge Baseline (2024) (8)	0.66[0.63, 0.73]			

**Figure 3:**
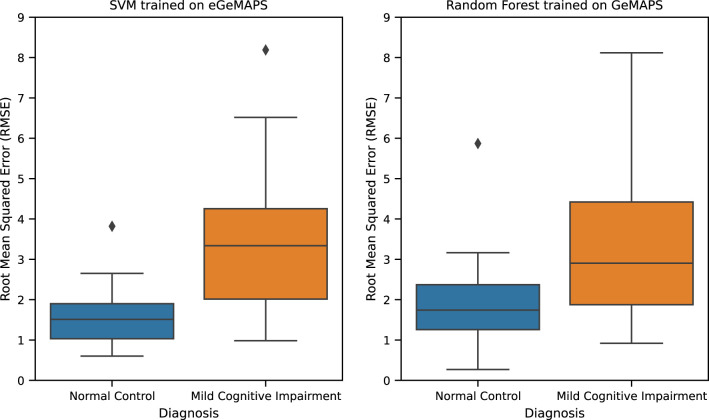
TAUKADIAL 2024 best regression models RMSE.

The best performing TAUKADIAL regression model (**Table 9**) is SVM trained with eGeMAPS functional features. It yields an RMSE score of 2.73 (±1.18) [2.16, 3.30]. Close to this performance is Random Forest trained with GeMAPS features. It yields an RMSE score of 2.77 (±1.21) [2.19, 3.35]. These models have a small performance difference with an effect size of *d* = 0.19 (*p* < 0.01). Hence, we also consider the two models to not be very different in terms of performance. Error analysis of these two best-performing models shows that they are biased against the MCI class (see **Figure 3**). In particular, an outlier above 8 was observed from the cross-validation RMSE of the SVM model, which is quite high given that the labels only range from 0 to 30. Despite this limitation, the model is slightly better than the reported baseline model in (8) with an RMSE score of 2.86 (no standard deviation reported) [2.5, 3.2].

**Table 9:** TAUKADIAL 2024 regression cross-validation results (*k* = 20).

Feature Set	SVM		Random Forest	
	RMSE (± std)	95% CI	RMSE (± std)	95% CI
ComParE 2016 Functionals	2.82 (1.12)	[2.28, 3.36]	2.83 (1.21)	[2.25, 3.42]
GeMAPS Functionals	2.78 (1.16)	[2.22, 3.33]	2.77 (1.21)	[2.19, 3.35]
eGeMAPS Functionals	2.73 (1.18)	[2.16, 3.30]	2.77 (1.24)	[2.18, 3.37]
Bag of Audio Words (n=500)	3.10 (1.32)	[2.47, 3.73]	3.02 (1.32)	[2.38, 3.65]
Challenge Baseline (2024) (8)	2.86 [2.5, 3.2]			

**Figure 4:**
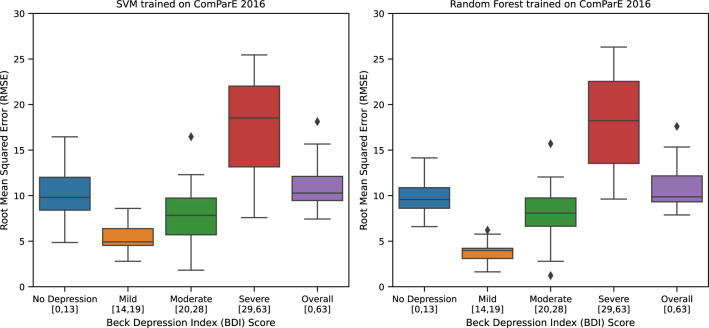
AVEC 2013 best regression models RMSE.

For AVEC dataset (**Table 10**), the best performing regression model is Random Forest trained with ComParE 2016 functional features. It has the lowest cross-validation RMSE score of 11.00 (± 2.25) [9.82,12.17]. This performance is slightly better than the reported challenge baseline in 2013 (4) with an RMSE score of 11.90 (no standard deviation and 95% CI reported). However, this performance is worse than the previously reported work (6) of the second and the last authors of this paper; their best performing model yields an RMSE score of 9.75 (no standard deviation and 95% CI reported). This performance difference is expected since no further improvements were attempted in our current replication experiment. This work nevertheless provides new information through error analysis; neither of the two cited works performed error analysis. In our analysis (see **Figure 4**), we reported the best performing model’s cross-validation RMSE scores on the four depression categories as defined in Beck Depression Index (BDI) (32): 1) no depression [0,13], 2) mild [14,19], 3) moderate [20,28], and 4) severe [29,63]. We also reported the overall RMSE score. We found through this analysis that the Random Forest model has the highest error in the severe depression group with a maximum recorded RMSE score above 25 units. This means that the predictions of the model in this group may be off by as much as 25 units in a 0 to 63 BDI scale. Even the model’s lowest median error of approximately 5 units in the mild depression group is not acceptable as an error this size could easily misclassify a sample from one depression category to another. This model needs performance improvement for all categories, especially on the severe depression group, which was an implicit objective of our 2019 study (6).

**Table 10:** AVEC 2013 regression cross-validation results (*k* = 20).

Feature Set	SVM		Random Forest	
	RMSE (± std)	95% CI	RMSE (± std)	95% CI
ComParE 2016 Functionals	11.14 (2.51)	[9.93, 12.35]	11.00 (2.45)	[9.82, 12.17]
GeMAPS Functionals	12.43 (3.25)	[10.87, 13.99]	11.35 (2.49)	[10.16, 12.55]
eGeMAPS Functionals	12.62 (3.16)	[11.10, 14.13]	11.08 (2.62)	[9.82, 12.34]
Bag of Audio Words (n=500)	11.49 (2.48)	[10.30, 12.68]	11.57 (2.29)	[10.47, 12.67]
Challenge Baseline (2013) (4)	11.90			
Tasnim & Stroulia (2019) (6)	9.75			

**Figure 5:**
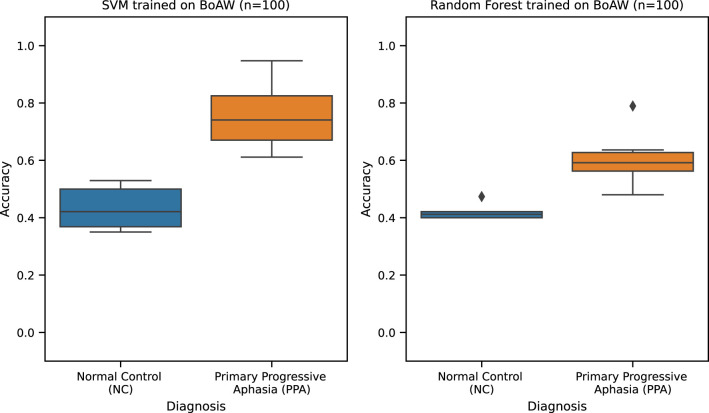
PPA best classification models accuracy.

For the PPA pilot study (**Table 11**), the best performing classification model is SVM trained with Bag-of-Audio-Word (*n* = 100) features. This model yields an F1 score of 0.42 (± 0.43) [0.14, 0.71], which is comparable to that of the second best performing model, i.e., Random Forest which was also trained with Bag-of-Audio-Word (n=100) features with an F1 score of 0.38 (± 0.38) [0.12, 0.63]. These two models have a small effect size of *d* = 0.19 (*p* < 0.01). Hence, we consider the two models to not be very different in terms of performance. However, as the preliminary results published in (26) were obtained using a subset of the dataset in this study, we could not make a fair comparison of these models with the baseline. Error analysis (see **Figure 5**) instead shows that both models have better performance in the Primary Progressive Aphasia (PPA) than the NC class. Moreover, Random Forest appears to be more stable with narrower accuracy score distributions for both classes. Despite these strengths, we strongly highlight that the high standard deviation and wide 95% CI of these models indicate weak and unstable performance. We believe that this limitation is due to the small sample size of this dataset with only 12 subjects (6 PPA and 6 NC). This variability undermines the stability and reliability of the model’s performance and prevents any immediate clinical applicability. We emphasize that these results should be considered preliminary and exploratory, representing a proof of concept. Validation on larger, independent cohorts with greater demographic and clinical diversity will be essential before drawing firm conclusions or pursuing clinical translation. As of this writing, the collection of a larger dataset is already ongoing.

**Table 11:** PPA classification cross-validation results (*k* = 12).

Feature Set	SVM		Random Forest	
	F1 (± std)	95% CI	F1 (± std)	95% CI
ComParE 2016 Functionals	0.37 (0.42)	[0.09, 0.64]	0.38 (0.43)	[0.09, 0.66]
GeMAPS Functionals	0.36 (0.43)	[0.08, 0.65]	0.36 (0.43)	[0.07, 0.64]
eGeMAPS Functionals	0.35 (0.41)	[0.08, 0.62]	0.35 (0.43)	[0.06, 0.63]
Bag of Audio Words (n=100)	0.43 (0.43)	[0.14,0.71]	0.38 (0.38)	[0.12, 0.63]

It is important to note here that the implementation of a comprehensive suite of metrics in the pipeline enables a thorough and consistent evaluation of all experimental configurations, which is, more often than not, missing from most publications in the literature. Such multi-perspective and consistent evaluation is essential for deciding future work in a manner that advances the state of the art.

## 5 Discussion

The work described in this paper is motivated by the realization that machine learning for speech analysis represents a substantial opportunity in the area of mental health. The scarcity of mental health services across the world, especially in developing countries, is driving the expansion of research and development of digital technologies for (tele)mental health. Key to delivering high-quality technology-enabled services is the ability to accurately assess and monitor an individual’s mental-health status and this is exactly the promise of speech-enabled machine-learning models.

As we have documented in the introduction section, this area is quite active and our work aims to systematize the software-engineering processes of this type of research, in order to increase its reproducibility and replicability and amplify its potential for use in the real world. To that end, we have developed a suite of configurable software pipelines that implement two speech analysis pipelines: 1) speech data preprocessing and feature extraction and 2) machine learning model training. We have evaluated our software pipelines by replicating three different experiments.

The results of these experiments demonstrate that our pipelines could replicate baseline results reported in the literature. In our error analysis, we further discussed the limitations of the models. This presentation of performance highlights the value of systematically saving experiment artifacts (e.g., validation predictions, selected features, optimal hyperparameters, models, and model performances) to perform further analysis. It is a straightforward practice that improves the traceability and verifiability of experimental results. Access to such artifacts, coupled with reusable code and configuration manifests, fosters transparency, which is essential to establish the reliability of results.

Our work to date has demonstrated the feasibility and usefulness of the current version of our software pipelines, while also highlighting several limitations. A limitation that we highlighted in our experiments is model bias. This bias is demonstrated through the error analysis that we performed, showing the models’ performance on different classes. For instance, the best performing model found for the TAUKADIAL dataset is biased against the “normal control” class. Moving forward, we aim to expand the range of supported tasks in our pipeline to include potential bias-mitigation strategies such as over- and under-sampling methods, e.g., SMOTE and random under-sampling. These methods will augment or reduce the number of training samples, respectively, to create balanced training sets.

We are also currently incorporating support for tasks such as deep spectrum feature extraction, deep-learning model training, and ensemble methods, including early fusion, where multiple feature types are combined before classification, and late fusion, where separate models trained on different data modalities or label sets are combined at the decision level. The deep-learning model training will be implemented as a separate pipeline as it requires more computational resources, but artifact preservation will follow the same approach described here. At the same time, we are further exploring the VoiceLab and TRESTLE tools to ensure that our pipeline covers their audio-processing functionalities.

Finally, we plan to deploy the pipelines in a secure cloud-based service platform. To this end, we are investigating different user interface designs that will allow users to configure, inspect, and compare their experimental pipelines more effectively.

## Data Availability

The data analyzed in this study is subject to the following licenses/restrictions: The datasets analyzed for this study can be found in the following links: (1) the TAUKADIAL 2024 Dataset: https://dementia.talkbank.org/TAUKADIAL/ and (2) the AVEC 2013 Dataset: Contact the authors of (4). The authors of the present work, Amable J. Cuervo and Enrique de la Cal, are members of the research team responsible for the design, collection, and deployment of the PPA dataset, which is being conducted under ethical approval code 2023.227 (33) at the University Hospital of Asturias (Spain). Accordingly, the team has full authorization to use this dataset within the scope of the current study, and no additional data-use agreements were necessary. Requests to access the dataset should be directed to Enrique A. de la Cal, delacal@uniovi.es.

## Footnotes

1
https://github.com/spotify/luigi

2
https://github.com/jiaaro/pydub

3
https://www.ffmpeg.org/general.html#File-Formats

4
https://github.com/wilsonchingg/logmmse

5
https://python-soundfile.readthedocs.io/en/0.11.0

6
https://github.com/wilsonchingg/logmmse

7
https://scikit-learn.org/stable/modules/generated/sklearn.model_selection.StratifiedGroupKFold.html

8
https://scikit-learn.org/stable/modules/generated/sklearn.preprocessing.StandardScaler.htm

9
https://scikit-learn.org/stable/modules/generated/sklearn.feature_selection.SelectKBest.html

10
https://citp.princeton.edu

11
https://docs.python.org/3/library/pickle.html
